# A protocol for mapping *Blastocystis *epidemiology and diagnostics from One Health perspective

**DOI:** 10.12688/openreseurope.20151.2

**Published:** 2025-06-18

**Authors:** Eylem Akdur Öztürk, Isabel Guadano-Procesi, Ana M. Figueiredo, Anja Godfrey, Eleni Gentekaki, Anastasios D. Tsaousis, David Carmena, Funda Dogruman-Al

**Affiliations:** 1 Department of Parasitology, Faculty of Medicine, Cukurova University, Sarçam, Adana, 01330, Turkey; 2 Department of Clinical Sciences and Translational Medicine, Faculty of Medicine, University of Tor Vergata, Rome, 00133, Italy; 3 Department of Biology and CESAM, University of Aveiro, Aveiro, 3810-193, Portugal; 4 School of Natural Sciences, University of Kent, Canterbury, England, CT2 7NZ, UK; 5 Department of Veterinary Medicine, School of Veterinary Medicine, University of Nicosia, Nicosia, 2414, Cyprus; 6 Parasitology Reference and Research Laboratory, Spanish National Centre for Microbiology,, Health Institute Carlos III, Majadahonda, Madrid, 28220, Spain; 7 Centre for Biomedical Research Network in Infectious Diseases (CIBERINFEC), Health Institute Carlos III, Madrid, 28029, Spain; 8 Division of Medical Parasitology, Department of Medical Microbiology, Faculty of Medicine,, Gazi University, Ankara, 06500, Turkey

**Keywords:** Prevalence, Subtype diversity, Standardisation, Europe, One Health, Human, Animal, Environment

## Abstract

*Blastocystis* is a globally prevalent gut protist colonising over a billion people worldwide, yet its epidemiology, transmission dynamics, and clinical significance remain underexplored. This protocol represents the first step of a large-scale effort to map *Blastocystis* epidemiology and diagnostic practices across Europe through the COST Action CA21105: *Blastocystis under One Health*. By assessing diagnostic methodologies across clinical, veterinary, and environmental sectors, this work sets the foundation for future research and standardisation. Here, we highlight key findings, challenges, and a roadmap for improving *Blastocystis* detection, ultimately influencing global health policies and microbial ecology studies.

## Plain language summary

Despite *Blastocystis* being discovered over 100 years ago, its role in health and disease remains unclear. Two issues that hinder our understanding of *Blastocystis*´ public health significance are the lack of standardised diagnostic methods and the poor knowledge of its epidemiology, particularly in animal and environmental reservoirs. To bridge this gap of knowledge, Working Group 1 of the COST Action CA21105: *Blastocystis* under One Health has developed two strategies. First, an online survey was implemented to assess *Blastocystis* awareness and how it is diagnosed, treated, and investigated across Europe, , to unify methods and provide evidence-based guidelines on diagnostics and research. Second, a scientific literature review was conducted to identify relevant studies on the prevalence and genetic diversity of *Blastocystis* across Europe to ascertain its geographical distribution patterns and transmission dynamics among human, animal, and environmental reservoirs.

## Introduction

*Blastocystis* is one of the most common intestinal protists in humans and animals, yet its biological significance, pathogenic potential, and epidemiological dynamics remain subjects of intense debate ^1^. Traditionally classified as a non-pathogenic organism, recent studies suggest *Blastocystis* may play a more complex role in gut microbiome homeostasis, immune modulation, and even disease associations ^2^. However, our understanding is hindered by inconsistencies in detection methodologies, lack of large-scale epidemiological data, and insufficient representation of diverse host populations.

Despite being detected in over a billion people worldwide, *Blastocystis* remains underrepresented in public health discussions, largely due to the absence of standardised diagnostic and reporting practices ^3^. The lack of consensus on its role—whether as a commensal, an opportunistic pathogen, or a marker of gut health—further complicates efforts to integrate it into mainstream microbiological surveillance. Most clinical laboratories lack protocols to detect *Blastocystis* systematically, leading to significant underreporting and misinterpretation of its presence ^3^.

Moreover, *Blastocystis* represents a unique model for One Health research due to its broad host range and zoonotic potential ^4^. The protist is found in diverse environments, from wastewater to livestock and wildlife reservoirs, raising concerns about potential transmission pathways and public health implications ^5– 7^. Yet, despite these concerns, studies on *Blastocystis* in environmental and animal samples remain scarce compared to human-focused research.

To address these challenges, COST Action CA21105 established working group 1 (WG1) with the primary objective of conducting the first Europe-wide mapping of *Blastocystis* epidemiology and diagnostics ^8^. This initiative seeks to:

1. **Standardise detection methodologies** to ensure reproducibility and comparability across studies, ultimately improving diagnostic accuracy.
2. **Assess the prevalence and subtype diversity of *Blastocystis* across Europe** by collecting data from clinical, veterinary, and environmental laboratories.
3. **Establish an open-access *Blastocystis* surveillance network**, enabling researchers and public health authorities to track trends in prevalence and subtype distribution.
4. **Facilitate interdisciplinary collaboration**, integrating microbiologists, parasitologists, clinicians, veterinarians, and environmental scientists to advance *Blastocystis* research.

## Methods

### Assessing how *Blastocystis* is detected across Europe

**Figure 1.**
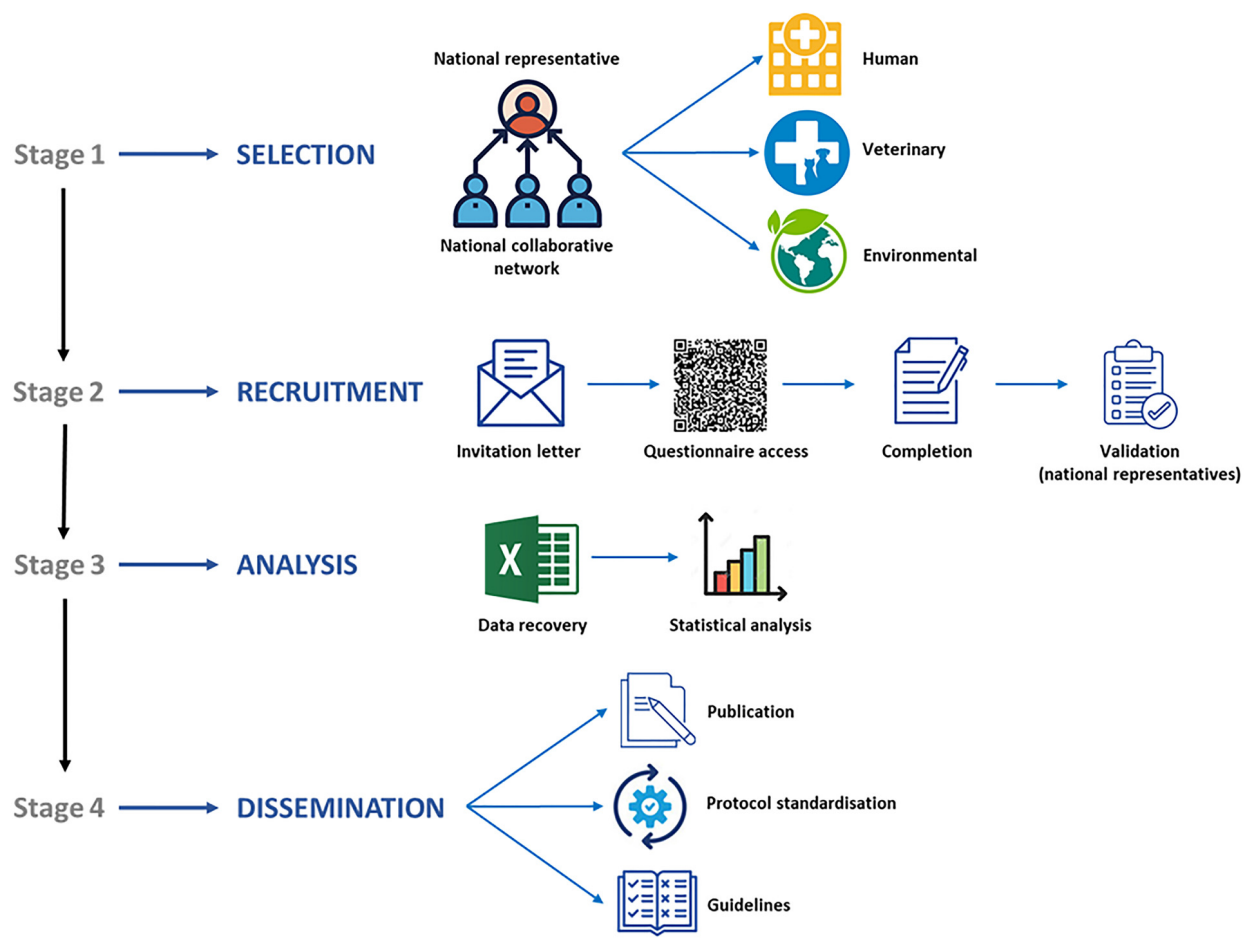
Diagram summarizing the main goals, working plan, and expected outcomes of the online survey `Mapping *Blastocystis* Epidemiology and Diagnostics´ developed by Working Group 1 within COST Action CA21105.

**Figure 2.**
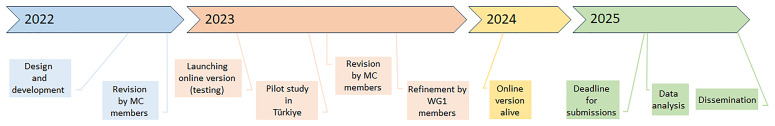
Timeline of the activities scheduled for the design, implementation, data collection, analysis, and dissemination of the online survey `Mapping *Blastocystis* Epidemiology and Diagnostics´ developed by Woking Group 1 within of COST Action CA21105.

An online survey (accessible at https://Blastocystis-cost.com/working-groups/wg1-Blastocystis-epidemiology-and-diagnostics/) has been created under WG1 of COST Action CA21105, titled “Mapping *Blastocystis* Epidemiology and Diagnostics”. This questionnaire aims to assess awareness of *Blastocystis* among European clinicians, veterinarians, and microbiologists, as well as to evaluate detection and genotyping methods across clinical, veterinary, and environmental institutions. The data collected will support the development of guidelines and standardised protocols, facilitating inter-laboratory comparisons, reproducibility, and knowledge exchange. The working plan to develop and implement this online survey is schematically presented in Figure 1. Detailed information about the procedures involved in the different stages of the project are as follows:

1. **Selection of participating countries**: To guarantee geographical representativity, the 41 COST full-member European countries (see https://www.cost.eu/about/members/) were initially targeted for inclusion in the survey.

2. **Estimation of participating institutions per country**: To ensure the representation of disciplines and research areas, institutions (clinical settings, academies, research and reference centres) working in the human, veterinary, and environmental fields were initially targeted for inclusion in the survey. The ideal number of participating institutions in the human and veterinary fields was proportionally estimated, taking into consideration the total human population of each country (one of each per one million people). Because of their scarcity, any identifiable institution in the environmental field was targeted for inclusion in the survey. This strategy, validated by an experienced epidemiologist, aimed to minimise potential bias due to demographic differences among countries.

3. **Design of the survey**: The timeline for completing this task is provided in Figure 2. WG1 leaders drafted the first version of the survey. It included multiple-choice and open-ended questions to allow respondents to clarify points or express their experiences and opinions more comprehensively. In its final version, the survey contained 62 questions divided into three main sections (see below) to ensure structured and systematic data collection and facilitate subsequent analysis. Depending on the number of sections to go through, the survey was designed to be completed in 15–20 min.

- Section 1 (questions 1.1 to 1.11 within questionnaire). Devoted to obtaining general information related to the respondents’ working institutions, main activity conducted, research scope, and methods used, including OMICs approaches.
- Section 2 (questions 2.1 to 2.21 within questionnaire). Questions in this section dealt with awareness and general knowledge on *Blastocystis*, perception on its pathogenic role, diagnostic procedures in place, and prescription of pharmacological treatment. Specifically intended for clinicians working in the human health care sector and aimed to evaluate approaches and practices on diagnosis, treatment, and research (if any) on *Blastocystis*. Non-clinical respondents were requested to move directly to Section 3.
- Section 3 (questions 3.1 to 3.30 within questionnaire). Devoted to collect detailed information on the methodologies employed for the diagnosis and genotyping of *Blastocystis*. It included questions to gauge respondent´s perception on how the development of standardised protocols could improve diagnostic, surveillance, and research efforts.

4. **Refinement**: Multiple rounds of revision were conducted first among the core group within WG1 and later among all WG1 members to guarantee that questions (i) were phrased in an accurate but concise manner without redundancy, (ii) had no ambiguous or misleading interpretations, and (iii) were easy to answer in a logic, intuitive way. The final version of the survey was formatted using the free web-based Google Forms tool for online dissemination and completion. This platform was chosen because of its user-friendly interface from any device with an internet connection, automatic saving, offline capabilities, automatic data aggregation into Google Sheets for real-time analysis and easy export, design capabilities, and simple sharing of response summaries, facilitating efficient data collection and collaboration for the whole group.

5. **Pilot study**: The survey was tested for comprehensibility, methodological, and operational issues in Türkiye. The country was chosen for this purpose due to its large and dedicated *Blastocystis* community. The experience gained during this process enabled the development of recommendations for the successful implementation of the survey in other countries, anticipating potential drawbacks. The Ethics Committee of Gazi University approved the survey and its procedures (Ref. 2023–950).

6. **Recruiting of participating institutions**: Identification of potentially suitable institutions, contacting, and invitation to participate in the survey were accomplished as follows:

- First, a national representative (NR) for each COST country was chosen among WG1 members on a voluntary basis. NRs were responsible, alone or in coordination with national collaborators of their choice, to identify the required numbers of participating institutions in their respective countries. They were also expected to disseminate and promote the survey in their professional networks and among relevant national academies, congresses and meetings, institutions, organisations, and scientific societies.
- Second, appropriate contacts at selected institutions were approached by NRs via an e-mail containing a formal invitation letter to participate in the survey. The letter briefly described the survey's goals and expected outcomes and provided access to the online survey via a hyperlink and a QR code. NRs were responsible for tracking the data collection process, identifying non-respondents and sending them gentle reminders to maximise participation.
- Third, efforts were also concentrated on countries with lower response rates by seeking support from a broader range of COST Action participants and reaching out to relevant societies, which distributed the survey, for example, by including it in their newsletters. Additionally, a list of further contacts in specific countries was compiled through online research. Participation incentives, including a 'refer a friend' card to help expand the reach, were created and distributed. Support was sought from the chair of the ESCMID Study Group for Clinical Parasitology, and the questionnaire was promoted at various presentations. Additional promotion of the survey was carried out by a WG1 young leader and the COST Project Officer attending the European Congress of Clinical Microbiology and Infectious Diseases (ESCMID) in Vienna (Austria) in April 2025 by engaging with a wider academic community and increasing visibility and international participation.

7. **Data collection and curation**: The whole process was thoroughly checked to guarantee the accuracy and consistency of the submitted responses. Missing or inconsistent data were identified and corrected. The generated full dataset is planned to be analysed in collaboration with an experienced epidemiologist.

### Assessing the prevalence and genetic diversity of *Blastocystis* across Europe

**Figure 3.**
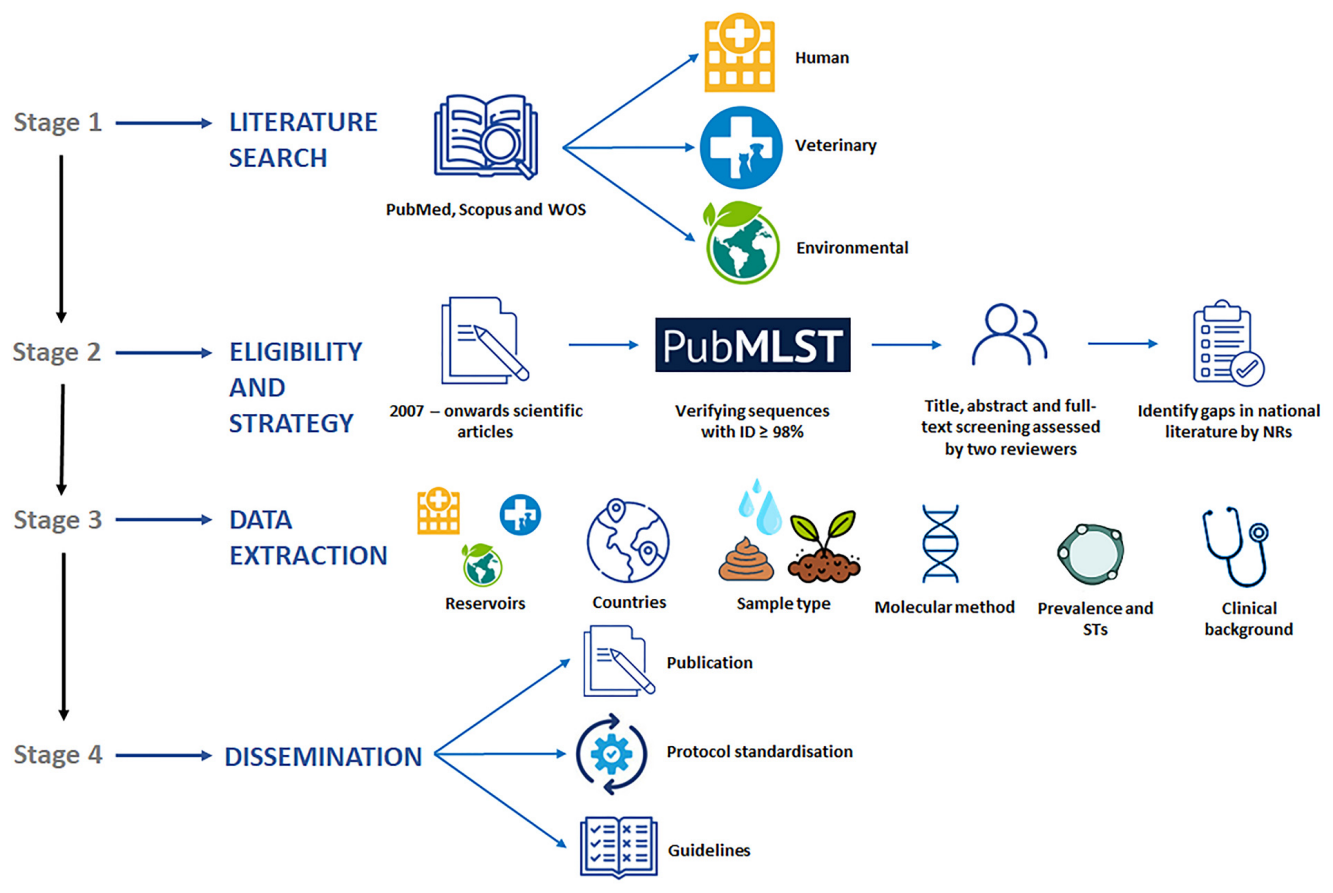
Diagram summarizing the main goals, working plan, and expected outcomes of the systematic review of the scientific literature on *Blastocystis* prevalence and molecular diversity published in Europe in the period 2007-present.

A comprehensive review of the relevant scientific literature will be carried out to determine the prevalence and subtype (ST) diversity and frequency of *Blastocystis* in the European scenario. The goal is to provide an integrated overview of the presence and transmission dynamics of *Blastocystis* among the human, animal (livestock, pet, wildlife), and environmental (water, soil, fresh produce) reservoirs to contribute to a better understanding of its public health significance and ecological role. The working plan to carry out this task is schematically presented in Figure 3. Detailed information about the procedures involved in the different stages of the project are as follows:

1. **Literature search**: A scientific literature review will be conducted using PubMed, Scopus, and Web of Science (WOS) databases to gather information on the prevalence and molecular data of *Blastocystis* across Europe. The selection process will follow a systematic approach primarily targeting studies that made a significant contribution to the field of *Blastocystis* epidemiology. The search strategy will include a combination of three search strings for each category (humans, animals and environment), combined with the Boolean operator “AND” to obtain only the intersection with the keyword “ *Blastocystis*” and each of the 41 European countries participating in the COST Action CA21105. Additional keywords such as “children” and “patients” will be included to capture studies focusing on infections in paediatric and clinical populations, allowing a better understanding of the potential public health significance of *Blastocystis*. For animals, in addition to the keywords “companion animals”, “pets”, “livestock”, “wild animals”, and “wildlife”, we will use the scientific genera of all European mammals ( *e.g.*, “ *Bos*”, “ *Capra*”, “ *Sus*”, “ *Ursus*”) to cover all wild/captive and domestic animals reported. To ensure a comprehensive assessment of environmental sources, additional terms to the keyword “environment” will be used, including “soil”, “water”, “food”, and “fresh produce”.

2. **Eligibility criteria**: Editorials, letters, commentaries, conference abstracts, narrative reviews, and non-peer-reviewed works ( *e.g.*, theses) will not be considered, but articles featured in their reference lists that did not appear in the three databases consulted will be assessed. Studies published in English will be prioritised, although exceptions will be made for relevant studies published in other languages. The search will cover the period 2007 (the year in which the first standardised nomenclature for *Blastocystis* STs of mammalian and avian origin was introduced ^9^) to present. Regardless of the initial detection method used, only studies offering ST-level identification adhering to the current nomenclature and providing sequence data with identity thresholds ≥98% (validated via PubMLST, see https://pubmlst.org/organisms/blastocystis-spp) will be retained to ensure consistency and comparability across datasets. Two independent reviewers will conduct screening and eligibility assessments. The first screening phase will involve reviewing the titles and abstracts of the studies and selecting those relevant to *Blastocystis*. The selected studies will also undergo a full-text screening. Potential discrepancies will be resolved by consulting a third reviewer.

3. **Strategy**: We will take advantage of the already established network of NRs created for the implementation of the online survey described above. In this case, NRs will be responsible for assisting in the identification of eligible publications from their respective countries, including grey literature. This approach will ensure that (i) collected data are accurate, robust, and reflective of the most recent epidemiological insights, and (ii) engagement with NRs will enable the identification of gaps or discrepancies in the existing national literature, enhancing the reliability and completeness of our dataset.

4. **Data extraction**: In addition to the author and year of publication, the following variables will be retrieved from eligible studies:

- **Reservoir**: The original source (human, animal, environmental) from which *Blastocystis* is identified. Age and gender will be recorded if available.
- **Country**: The geographic location where the study was carried out.
- **Type of sample**: The specific sample (faecal matter, cultured isolate) or matrix (water, soil, food) employed for molecular testing.
- **Molecular method(s) used for detection purposes**: The type of PCR (direct, nested, real-time, other) used in the study.
- **Prevalence**: The frequency of individuals or animals in the study population carrying *Blastocystis*, expressed as a percentage or ratio.
- **Subtype**: The *Blastocystis* subtype (ST) (as determined by Sanger or next-generation- amplicon sequencing methods) reported in the study.
- **Clinical background**: The symptoms and co-infections with other parasites reported in the study.

## Results

We expect to provide the most comprehensive and updated picture, under the One Health umbrella, of the epidemiology of *Blastocystis* in Europe to date. The obtained results will have a significant impact in (i) determining current limitations of the molecular tools available for detection and genotyping purposes of *Blastocystis*, (ii) assessing public health implications, particularly concerning the potential zoonotic transmission of *Blastocystis* and the identification of dominant/emerging STs, (iii) determining geographical differences in *Blastocystis* prevalence and ST, a potentially useful information to assist policymakers and public health authorities in the design and implementation of targeted interventions, and (iv) identifying gaps in current knowledge and future research directions.

## Discussion and conclusion

This study protocol is a landmark in *Blastocystis* research, providing the conceptual framework for diagnostic standardisation and epidemiological mapping. WG1’s work will shape the future of *Blastocystis* investigations in Europe and beyond by addressing methodological disparities and fostering a collaborative research environment. Implementing standardised protocols and expanded data sharing will significantly enhance the accuracy of *Blastocystis* diagnosis and research, influencing global One Health policies and microbial surveillance schemes. Moreover, this initiative lays the groundwork for integrating *Blastocystis* into broader microbial ecology and clinical microbiology paradigms, ensuring its recognition as an important gut microbe with implications for health and disease.

## Ethics and consent

This study was conducted in accordance with the Declaration of Helsinki, and approved by the Ethics Committee of Gazi University (Approval number: 2023–950, Date: August 02, 2023). As there was no involvement with patients within the scope of this study, confidential or sensitive information that would require protection was not managed. Therefore, signed informed consent from participants in the online survey were deemed not necessary.

An invitation letter was sent to the National Representatives (NRs) to disseminate the survey through their respective national associations, institutes, and scientific/academic networks.

The invitation letter clearly outlined the purpose and significance of the survey within the context of the COST Action. The letter provided a link to the survey, and participants who clicked on the link were presented with the statement “Personal information collected from this survey will not be disclosed under any circumstances” at the beginning of the survey form.

Participation in the survey was expected to be entirely voluntary, limited to adult researchers/participants. There was no obligation to participate, and the decision to complete the survey was left solely to the discretion of the respondents. Signed informed consents from participants in the online survey were deemed not necessary. Our study proposal and methodology were reviewed and approved by the Gazi University Ethics Committee.

## Data Availability

No data associated with this article
