# Supplementary material for: A protocol for mapping Blastocystis epidemiology and diagnostics from One Health perspective
Source: Open Res Eur. 2025 Jun 30;5:133. Originally published 2025 May 16. [Version 3] doi: 10.12688/openreseurope.20151.3 (PMC12171710; doi:10.12688/openreseurope.20151.3)
Supplement: Peer review history

# Reviewer report: Reviewer response for version 3

**DOI:** 10.21956/openreseurope.22434.r56289

**Published:** 2025-07-12

**Authors:** Małgorzata Lepczyńska

**Affiliations:** 1 Department of Medical Biology, School of Public Health, Collegium Medicum, University of Warmia and Mazury, Żołnierska, Poland

I have no further comments to make concerning this manuscript. I recommend Indexing.

Is the study design appropriate for the research question?

Yes

Is the rationale for, and objectives of, the study clearly described?

Yes

Are sufficient details of the methods provided to allow replication by others?

Yes

Are the datasets clearly presented in a useable and accessible format?

Not applicable

Reviewer Expertise:

medical microbiology, medical parasitology

I confirm that I have read this submission and believe that I have an appropriate level of expertise to confirm that it is of an acceptable scientific standard.

# Reviewer report: Reviewer response for version 2

**DOI:** 10.21956/openreseurope.22400.r55693

**Published:** 2025-06-24

**Authors:** Małgorzata Lepczyńska

**Affiliations:** 1 Department of Medical Biology, School of Public Health, Collegium Medicum, University of Warmia and Mazury, Żołnierska, Poland

I have carefully read the revised version of the manuscript. In light of improvements, I find the manuscript to be a valuable contribution to the literature on *Blastocystis* research, and I recommend its acceptance. I have no further comments.

Is the study design appropriate for the research question?

Yes

Is the rationale for, and objectives of, the study clearly described?

Yes

Are sufficient details of the methods provided to allow replication by others?

Yes

Are the datasets clearly presented in a useable and accessible format?

Not applicable

Reviewer Expertise:

medical microbiology, medical parasitology

I confirm that I have read this submission and believe that I have an appropriate level of expertise to confirm that it is of an acceptable scientific standard.

# Reviewer report: Reviewer response for version 1

**DOI:** 10.21956/openreseurope.21797.r54632

**Published:** 2025-06-16

**Authors:** Arutchelvan Rajamanikam

**Affiliations:** 1 University Malaya, Kuala Lumpur, Malaysia

The report is comprehensive and addresses the major concern in *Blastocystis *sp. research for decades. The report is based on secondary data from the databases. Hence, it is important to take into consideration the confounders in the studies included. 

 The impacts were clearly communicated and it definitely bridges the gaps in *Blastocystis *research.

Is the study design appropriate for the research question?

Yes

Is the rationale for, and objectives of, the study clearly described?

Yes

Are sufficient details of the methods provided to allow replication by others?

Yes

Are the datasets clearly presented in a useable and accessible format?

Yes

Reviewer Expertise:

Molecular parasitology, gut microbiology, host-parasite interaction.

I confirm that I have read this submission and believe that I have an appropriate level of expertise to confirm that it is of an acceptable scientific standard.

# Reviewer report: Reviewer response for version 1

**DOI:** 10.21956/openreseurope.21797.r54630

**Published:** 2025-06-16

**Authors:** Renzo S Salazar-Sánchez

**Affiliations:** 1 IMBI, University Hospital Heidelberg (Ringgold ID: 27178), Heidelberg, Baden-Württemberg, Germany

The development of studies within the One Health framework offers a holistic, proactive, and sustainable strategy to bridge critical knowledge gaps in the understanding of Blastocystis, a globally distributed protozoan found across multiple hosts. The authors propose an ambitious and timely study aimed at mapping the epidemiology of Blastocystis from a One Health perspective.

 One of the main challenges lies in coordinating across multiple institutions to encompass the key components of the One Health approach—human, animal, and environmental health. Nevertheless, the authors have outlined various activities intended to foster interdisciplinary collaboration and support integrated research efforts and data obtained.

 In relation to data collection via the proposed questionnaire, several broad aspects require more detailed explanation. Without careful design, there's a risk of introducing bias by excluding centers with limited access to sequencing or molecular diagnostics. To minimize this potential bias across different countries and settings—whether clinical, environmental, or animal—the authors propose the involvement of epidemiological experts. However, a more robust and clearly defined strategy is needed for data harmonization and curation, ideally led by a multidisciplinary team that includes clinicians, veterinarians, and environmental epidemiologists.

 While the implementation of this study protocol has the potential to significantly advance our understanding of Blastocystis and inform One Health policies, certain methodological details must be addressed to ensure a truly integrative and comprehensive approach.

Is the study design appropriate for the research question?

Yes

Is the rationale for, and objectives of, the study clearly described?

Yes

Are sufficient details of the methods provided to allow replication by others?

Partly

Are the datasets clearly presented in a useable and accessible format?

Yes

Reviewer Expertise:

Public health, Zoonotic and infectious diseases, Parasitology, and Molecular epidemiology.

I confirm that I have read this submission and believe that I have an appropriate level of expertise to confirm that it is of an acceptable scientific standard.

# Reviewer report: Reviewer response for version 1

**DOI:** 10.21956/openreseurope.21797.r54634

**Published:** 2025-06-16

**Authors:** Małgorzata Lepczyńska

**Affiliations:** 1 Department of Medical Biology, School of Public Health, Collegium Medicum, University of Warmia and Mazury, Żołnierska, Poland

This manuscript presents a creating process of valuable protocol for conducting the mapping of *Blastocystis* epidemiology and diagnostics in Europe. The focus of this topic is extremely relevant, considering the current ambiguities in *Blastocystis* detection methods, gaps in epidemiological data, and doubtful clinician awareness of *Blastocystis* prevalence and pathogenicity. The authors present a scheme of protocol that aims both to compare the practices and explanatory criteria used by diagnostic and research laboratories across Europe for isolation, identification, and subtyping of *Blastocystis* and to investigate the epidemiology of *Blastocystis* in Europe, using newly established generic guidelines and criteria.

 To achieve these goals, the authors developed two strategies:

 1. Assessing the methods of *Blastocystis* detection—by online survey designed to assess awareness of *Blastocystis* among European clinicians, veterinarians, and microbiologists, as well as to evaluate detection and genotyping methods across various research and healthcare institutions. This survey adequately covers the different details of aspects that need to be investigated in line with the research objective of assessing the detection methods across various laboratories as well as reporting the intensity of *Blastocystis* cases. The questionnaire is well thought out, and the division into three parts containing relevant questions makes it more transparent. Given the various existing and confusing protocols on this topic and the difficulty of standardization, I consider this idea extremely important.

 2. Assessing the prevalence and genetic diversity of *Blastocystis* in European countries—by scientific literature review from the last 20 years to identify relevant studies on the geographical distribution patterns of *Blastocystis* subtypes across Europe and its transmission dynamics among human, animal, and environmental reservoirs. The authors very carefully choose the inclusion and exclusion criteria of the reviewed literature.

 The data collected in this way will finally support the development of guidelines and standardized protocols, improving methodology as well as facilitating communication and knowledge exchange.

 Each part of the manuscript presents the content in a clear, orderly, and rigorous manner; the state of the art is well reflected. The introduction offers an explicit description of the study’s aims while effectively linking the discrepancies in methodology of *Blastocystis* detection and underreporting of its presence to the broader context of clinicians’ unawareness.

 The presence of diagrams summarizing the main goals, working plan, and expected outcomes of the online survey, as well as the systematic review of the scientific literature on *Blastocystis* in this article, strengthens its understanding and demonstrates the protocol’s effectiveness.

 Overall, the study protocol will represent a good step toward *Blastocystis* detection worldwide. The manuscript specifies what the study aims to achieve with this protocol.

 In general, the article is well written and structured. The methodology is presented in detail. In light of these substantial topics discussed, I find the manuscript to be a valuable contribution to the research on *Blastocystis*, and I recommend its acceptance.

Is the study design appropriate for the research question?

Yes

Is the rationale for, and objectives of, the study clearly described?

Yes

Are sufficient details of the methods provided to allow replication by others?

Yes

Are the datasets clearly presented in a useable and accessible format?

Not applicable

Reviewer Expertise:

medical microbiology, medical parasitology

I confirm that I have read this submission and believe that I have an appropriate level of expertise to confirm that it is of an acceptable scientific standard.

# Reviewer report: Reviewer response for version 1

**DOI:** 10.21956/openreseurope.21797.r54626

**Published:** 2025-06-02

**Authors:** Supaluk Popruk

**Affiliations:** 1 Mahidol University, Bangkok, Thailand

When conducting studies involving humans, animals, and environmental sources across different regions or countries, the selection of clinical settings, academic institutions, and research or reference centers must be made with careful consideration to minimize potential bias.

 It would be helpful to clarify how the questions were distributed to participants in each group and what criteria were used to guide this process. Furthermore, some clinical laboratories may only provide binary diagnostic results—indicating either the presence or absence of infection—without the capacity to identify subtypes. In contrast, academic and research institutions, as well as national and international reference centers, are typically equipped with advanced molecular tools that allow for detailed subtype identification, including sequencing.

 It is also unclear whether Section 2 includes questions measured on an ordinal scale. If so, it is recommended that clear interpretation criteria be established to ensure consistency and validity in the analysis.

 Additionally, the author may consider including further keywords such as *rodents*, *birds*, *non-human primates*, and *wastewater* to improve the comprehensiveness and discoverability of the study.

Is the study design appropriate for the research question?

Yes

Is the rationale for, and objectives of, the study clearly described?

Yes

Are sufficient details of the methods provided to allow replication by others?

Yes

Are the datasets clearly presented in a useable and accessible format?

Yes

Reviewer Expertise:

Parasitology

I confirm that I have read this submission and believe that I have an appropriate level of expertise to confirm that it is of an acceptable scientific standard.
